# Bone Marrow Homeostasis Is Impaired via JAK/STAT and Glucocorticoid Signaling in Cancer Cachexia Model

**DOI:** 10.3390/cancers13051059

**Published:** 2021-03-02

**Authors:** Jinyeong Yu, Sanghyuk Choi, Aran Park, Jungbeom Do, Donghyun Nam, Youngjae Kim, Jinok Noh, Kil Yeon Lee, Chi Hoon Maeng, Ki-Sook Park

**Affiliations:** 1 Graduate School of Biotechnology, Kyung Hee University, Yongin 17104, Korea; jinyeong90@khu.ac.kr (J.Y.); sanghyuk@khu.ac.kr (S.C.); arvi2114@khu.ac.kr (A.P.); 2 Department of Biomedical Science and Technology, Graduate School, Kyung Hee University, Seoul 02447, Korea; wndqja9355@naver.com (J.D.); ndh0721@naver.com (D.N.); kyj3002@naver.com (Y.K.); rjo8853@khu.ac.kr (J.N.); 3 Department of Surgery, College of Medicine, Kyung Hee University, Seoul 02447, Korea; kilyeonlee@khu.ac.kr; 4 Department of Internal Medicine, College of Medicine, Kyung Hee University, Seoul 02447, Korea; mchihoon@khu.ac.kr; 5 East-West Medical Research Institute, Kyung Hee University, Seoul 02447, Korea

**Keywords:** cancer cachexia, bone marrow, mesenchymal stem cells, JAK, STAT3, glucocorticoid

## Abstract

**Simple Summary:**

Cancer cachexia is a systemic inflammatory disease characterized by the loss of muscle and fat and occurs in 50–80% of cancer patients. In cancer cachexia, the tumor tissues interact with other tissues and organs using secretory factors. Differentiated immune cells from hematopoietic stem cells (HSCs) of the bone marrow contribute to the systemic inflammation and may be affected by these intertissue interactions. However, the significant changes that occur in the bone marrow and the underlying mechanisms are still unclear. Here, we investigated the effects of cancer cachexia on bone and stem cells that reside in the bone marrow using a lung cancer cachexia animal model. Cancer cachexia induces bone loss and impairs the properties of the bone marrow mesenchymal stem cells via JAK/STAT and glucocorticoid signaling. Our findings provide new insights for developing a novel therapeutic strategy for cancer cachexia.

**Abstract:**

Cancer cachexia is a multifactorial systemic inflammation disease caused by complex interactions between the tumor and host tissues via soluble factors. However, whether cancer cachexia affects the bone marrow, in particular the hematopoietic stem cells (HSCs) and mesenchymal stem cells (MSCs), remains unclear. Here, we investigated the bone marrow and bone in a cancer cachexia animal model generated by transplanting Lewis lung carcinoma cells. The number of bone marrow mononuclear cells (BM-MNCs) started to significantly decrease in the cancer cachectic animal model prior to the discernable loss of muscle and fat. This decrease in BM-MNCs was associated with myeloid skewing in the circulation and the expansion of hematopoietic progenitors in the bone marrow. Bone loss occurred in the cancer cachexia animal model and accompanied the decrease in the bone marrow MSCs that play important roles in both supporting HSCs and maintaining bone homeostasis. Glucocorticoid signaling mediated the decrease in bone marrow MSCs in the cancer cachectic environment. The cancer cachexia environment also skewed the differentiation of the bone marrow MSCs toward adipogenic fate via JAK/STAT as well as glucocorticoid signaling. Our results suggest that the bone loss induced in cancer cachexia is associated with the depletion and the impaired differentiation capacity of the bone marrow MSCs.

## 1. Introduction

Cancer cachexia is a systemic disease that is characterized by chronic inflammation and progressive weight loss associated primarily with the depletion of fat tissue and skeletal muscle [1,2]. The prevalence of cancer cachexia is dependent on the tumor type and is estimated to be as high as 50–80% in cancer patients [1,3]. Cancer cachexia leads to mortality in 20% of cancer patients and is clinically associated with metastasis; the severity of cancer cachexia is often not related to tumor size or stage [3,4,5]. However, the etiology of cancer cachexia is not clearly understood and the mechanisms for the initiation of the depletion in fat and muscles in cancer patients remain to be elucidated to improve diagnosis and treatment.

In cancer cachexia, both tumor cells and tumor-activating immune cells express proinflammatory cytokines, including tumor necrosis factor (TNF)-α, interleukin (IL)-1, IL-6, IL-8, and interferon (IFN)γ, all of which systemically affect various organs such as the brain, liver, muscle, and fat [1,2,6]. For example, C-reactive protein (CRP), a diagnostic marker of cancer cachexia, is synthesized in the liver in response to systemic IL-6 [7]. Indeed, although most of the cytokines are considered as biomarkers of cancer cachexia along with CRP, they need cautious validation [7,8]. Anticytokine therapies are considered for the treatment of cancer cachexia. The blocking of the IL-6 receptor with suramin alleviated several effects of cachexia in an experimental model of cancer cachexia [9,10]. Thalidomide, an inhibitor of TNF-α synthesis with anti-inflammatory properties, attenuates weight loss in patients with cancer cachexia [11]. However, none of the anticytokine therapies that represent a promising treatment strategy for cancer cachexia have been authorized for the clinical treatment of the disease at present.

Glucocorticoid has important roles in the development of cancer cachexia [1,12]. Serum glucocorticoid level is higher in cancer patients compared to that in healthy individuals [1]. Indeed, IL-6, associated with proinflammatory immune response, activates JAK/STAT signaling and drives the secretion of glucocorticoid that causes loss of muscle, a typical characteristic of cancer cachexia [2,13]. Considering that muscle loss in a muscle atrophy animal model was mediated by glucocorticoid signaling via the forkhead box type O (FOXO) transcription factors in skeletal muscles [14], muscle wasting in cancer cachexia is also mediated by glucocorticoid signaling in skeletal muscles [12,15]. Glucocorticoid and synthetic glucocorticoid are paradoxically used as adjuvants in cancer cachexia treatment because they alleviate symptoms, such as appetite loss, although their use is limited to the end-stage of the disease on a short term basis (a few weeks) [12].

Tumor induces myeloid skewing and an increase in the production of myeloid cells including myeloid-derived suppressor cells (MDSCs) [16,17] as myelopoiesis is enhanced in response to inflammation and physiological aging [18,19]. It has been proposed that the expansion of MDSCs partially contributes to the development of cancer cachexia [20,21]. The expansion of myeloid cells is induced by tumor-mediated changes in the hematopoiesis of the hematopoietic stem cells (HSCs) [22] that reside in the bone marrow within a specialized microenvironment, HSC niche [23,24]. Hematopoiesis, cellular proliferation, and HSC activation are regulated extrinsically by the HSC niche as well as intrinsically [23,24]. Mesenchymal stem cells (MSCs) are multipotent stem cells derived from the bone marrow that can be differentiated into several different cell types, such as osteoblasts and adipocytes [25], and constitute the major cellular components of the HSC niche [23]. Therefore, bone marrow MSCs play essential roles in both the maintenance of bone mass and the regulation of HSC hematopoiesis. Inflammation and physiological aging regulate HSC hematopoiesis via the remodeling of the niche [18,19]. Although proinflammatory cytokines regulate the differentiation of MSCs into osteoblasts and adipocytes in vitro, contradictory results were observed in an inflammatory environment; the pretreatment of MSCs with inflammatory cytokines was reported to enhance or suppress the osteogenic differentiation [26,27,28]. Proinflammatory cytokines, including IL-6, IFN-γ, TNF-α, and IL-17, also modulate the proliferation and motility of MSCs in vitro [29]. Similarly, glucocorticoid also plays essential roles in modulating the characteristics of the bone marrow MSCs. Glucocorticoid and dexamethasone, a potent synthetic glucocorticoid [30,31], cause osteoporosis and nontraumatic osteonecrosis [32,33]. Dexamethasone induces the adipogenic [25] and osteogenic [34] differentiation of MSCs. Moreover, it dose-dependently decreases the colony number of bone marrow MSCs derived from old and young mice [35]. However, whether cancer cachexia induced by tumor development affects bone marrow MSCs that regulate bone mass and bone marrow hematopoiesis remains unknown. In the present study, we used the Lewis lung carcinoma (LLC)-induced cancer cachexia animal model to show that cancer cachexia impairs the colony-forming ability and the differentiation potential of bone marrow MSCs. We further investigated the molecular mechanisms underlying this impairment in the animal model.

## 2. Results

### 2.1. LLC-Induced Cancer Cachexia in Mice

**Figure 1:**
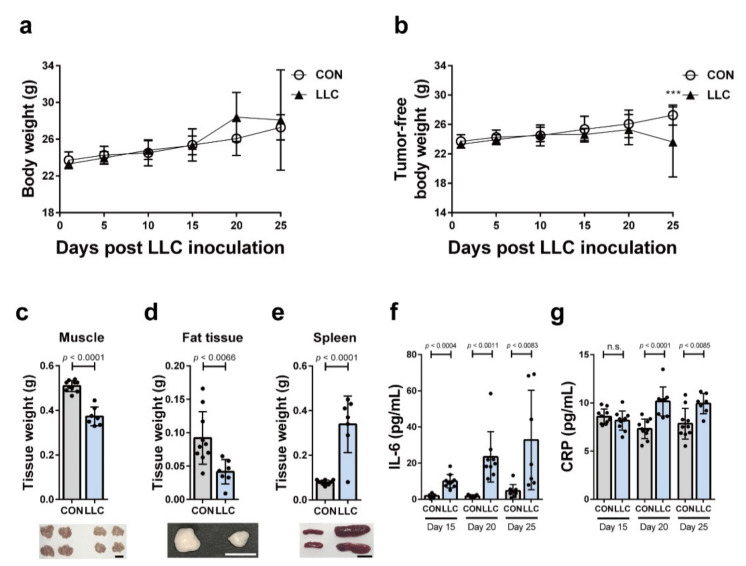
Cancer cachectic markers were quantified in LLC-bearing mice. (**a**,**b**) Body weights or tumor-free body weights of LLC-bearing mice (LLC) were measured every five days after LLC inoculation. As the control, body weights or tumor-free body weights of sham-operated mice (CON) were measured (*n* = 7–10 mice per group at each time point; *** *p* < 0.0002, two-way ANOVA). Muscle tissue (**c**), fat tissue (**d**), and spleen (**e**) of LLC-bearing mice (LLC) and the control (CON) were weighed at 25 days postinoculation of LLC cells or the control mice at 25 days post-sham-operation (*n* = 7–10 mice per group). (**f**,**g**) Serum level of IL-6 and CRP was measured at 15, 20, or 25 days postinoculation of LLC cells (LLC) or post-sham-operation (CON) (*n* = 7–10 mice per group at each time point). Data are presented as the mean ± SD and *p* values were obtained by *t*-tests. The scale bars indicate 1 cm.

We used a LLC-induced cancer cachexia model to investigate if cancer cachexia affects bone marrow homeostasis. LLC cells were subcutaneously injected into the flank of syngeneic C57BL/6J mice. Gender- and age-matched control mice received a sham operation. LLC cells formed tumors in the experimental mice and the body weight of LLC-bearing mice did not significantly differ from that of the control mice up to 25 days after the inoculation of LLC cells (Figure 1a). However, the tumor-free body weight of LLC-bearing mice was significantly lower than that of the control 25 days postinoculation of LLC (Figure 1b). To examine if the decrease in the tumor-free body weight of the tumor-bearing mice was associated with the loss of muscle and fat, we weighed the inguinal adipose tissue and tibial muscles 25 days after LLC cells inoculation. Both muscle and fat weight were lower in LLC-bearing mice compared to that in the controls (Figure 1c,d). Notably, the spleen weight in the LLC-bearing mice was greater compared to that in the controls (Figure 1e). Systemic inflammation is one of the main characteristics of cancer cachexia. Interleukin-6 (IL-6) is a proinflammatory cytokine driving systemic inflammation that leads to cancer cachexia. IL-6 induces the synthesis of CRP, a sensitive marker of cancer cachexia [7] as well as inflammation [36,37]. Compared to the control mice, the circulating IL-6 levels increased by day 15 postinoculation of LLC in the LLC-bearing mice and continued to increase up to day 25 (Figure 1f). Serum CRP levels were also significantly increased compared to those in the control mice (Figure 1g). These results indicate that LLC-bearing mice showed cancer cachectic characteristics, including loss of body weight, wasting of muscle and fat, and systemic inflammation.

### 2.2. Cancer Cachexia Leads to Myeloid Skewing in the Circulation and Increases Hematopoietic Progenitors of the Bone Marrow

**Figure 2:**
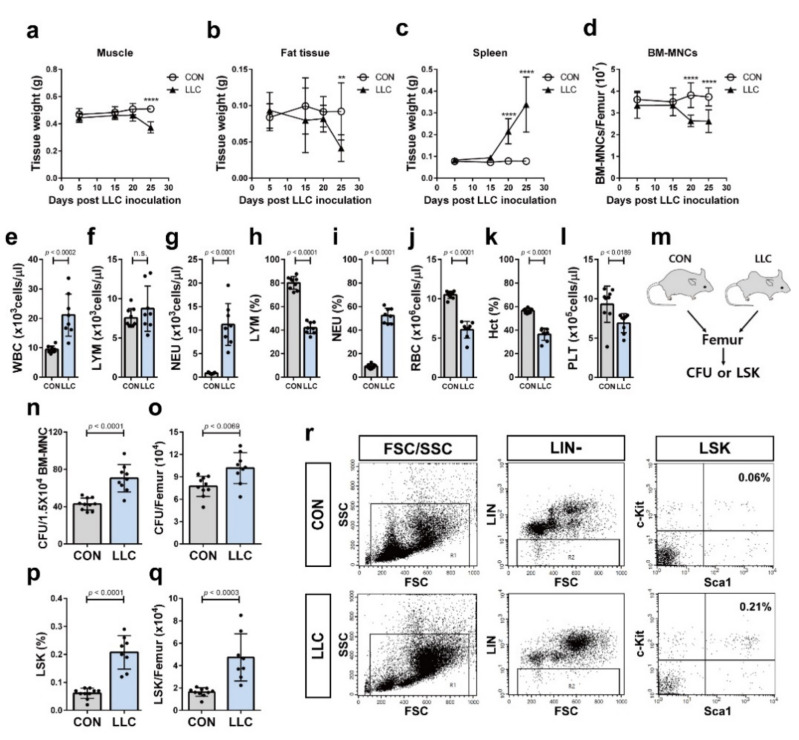
LLC cancer cachexia decreased the number of bone marrow mononuclear cells and induced myeloid skewing in the circulation. The weight of muscle tissue (**a**), fat tissue (**b**), and spleen (**c**) and the number of bone marrow mononuclear cells (BM-MNCs) (**d**) of LLC-bearing mice (LLC) and the control (CON) were measured every five days after LLC inoculation or sham-operation, respectively (*n* = 7–10 mice per group at each time point; **** *p* < 0.0001, ** *p* < 0.005, two-way ANOVA). Frequencies of white blood cells (WBC), lymphocytes (LYM), and neutrophils (NEU) (**e**–**g**), the percentages of lymphocytes (LYM) and neutrophils (NEU) (**h**,**i**), the number of red blood cells (RBC) (**j**), the percentage of hematocrit (Hct) (**k**), and the number of platelets (PLT) (**l**) were analyzed in peripheral blood of LLC cancer cachectic mice at 25 days postinoculation of LLC cells (LLC) (*n* = 8 mice) or the control mice at 25 days post-sham-operation (CON) (*n* = 9 mice). (**m**) Schematic representation of colony forming unit (CFU) assay and analysis of Lin-, Sca1+ and c-Kit+ (LSK) cells using femurs of LLC-bearing mice at 25 days postinoculation of LLC cells (LLC) or the control mice at 25 days post-sham-operation (CON). Frequency of CFU (**n**) or the number of CFU per femur (**o**) were analyzed in LLC cancer cachectic mice (LLC, *n* = 9 mice) or the control mice (CON, *n* = 10 mice). Percentage of LSK cells (**p**) or the number of LSK cells per femur (**q**) were defined by flow cytometry in LLC cancer cachectic mice (*n* = 8 mice) or the control mice (*n* = 10 mice). (**r**) Representative flow cytometric pattern (FSC, forward scatter; SSC, side scatter; LIN-, lineage negative; LSK, Lin-, Sca1+ and c-Kit+). Data are presented as the mean ±  SD (*p* values were obtained by *t*-tests. n.s.; not significant).

Spleen enlargement is associated with tumor-induced increase in myeloid cells [20,38,39,40]. Solid tumor cells crosstalk with the HSCs of the bone marrow leading to hematopoiesis and myeloid skewing of the HSCs [22,41]. We evaluated the changes in the number of bone marrow mononuclear cells (BM-MNCs) as well as the weights of the muscle, fat, and spleen as cancer cachexia progressed in the LLC-bearing mice. We observed that the spleen weight and the number of BM-MNCs were affected before the reduction in muscle and fat weight in the LLC-bearing mice (Figure 2a–d; 20 days postinoculation of LLC vs. 25 days). The number of BM-MNCs was significantly decreased in LLC-bearing mice before the decrease in muscle and fat weight began (Figure 2a,b,d; 20 days postinoculation of LLC vs. 25 days). To investigate if LLC-bearing mice showed myeloid skewing, peripheral blood of these cancer cachectic mice was analyzed. LLC-bearing mice showed a significant increase in the complete white blood cell and neutrophil counts compared to those in the control mice; however, their lymphocyte counts were not changed (Figure 2e–g). The percentage of lymphocytes in LLC-bearing mice significantly decreased (Figure 2h). In contrast, the percentage of neutrophils in LLC-bearing mice significantly increased (Figure 2i). LLC-bearing mice significantly decreased red blood cells, the platelets, and hematocrit value (Figure 2j–l). These results suggest that the myeloid skewing is associated with the decrease in the number of BM-MNCs of LLC-bearing mice. Tumors were shown to induce profound remodeling of the bone marrow hematopoiesis and increase the population of bone marrow hematopoietic progenitors [22,41]. Indeed, LLC-bearing mice exhibited a higher number of colony forming units (CFUs) from hematopoietic progenitors of the bone marrow than the control mice (Figure 2m–o), further indicating an increase in hematopoiesis. Hematopoietic progenitors in the bone marrow were analyzed by flow cytometry. Lin-, Sca1+, and c-Kit+ cells (LSK) of the mouse bone marrow contain all the hematopoietic progenitors [42,43]. In the present study, the percentage and yield of LSK were enhanced in the bone marrow of LLC-bearing mice, compared to those of the control mice (Figure 2p–r). Overall, these results suggest that the distant tumor cells in LLC-bearing mice systemically affect hematopoiesis of the bone marrow HSCs.

### 2.3. Cancer Cachexia-Induced Bone Loss Is Associated with the Decrease in the Population of Bone Marrow MSCs

**Figure 3:**
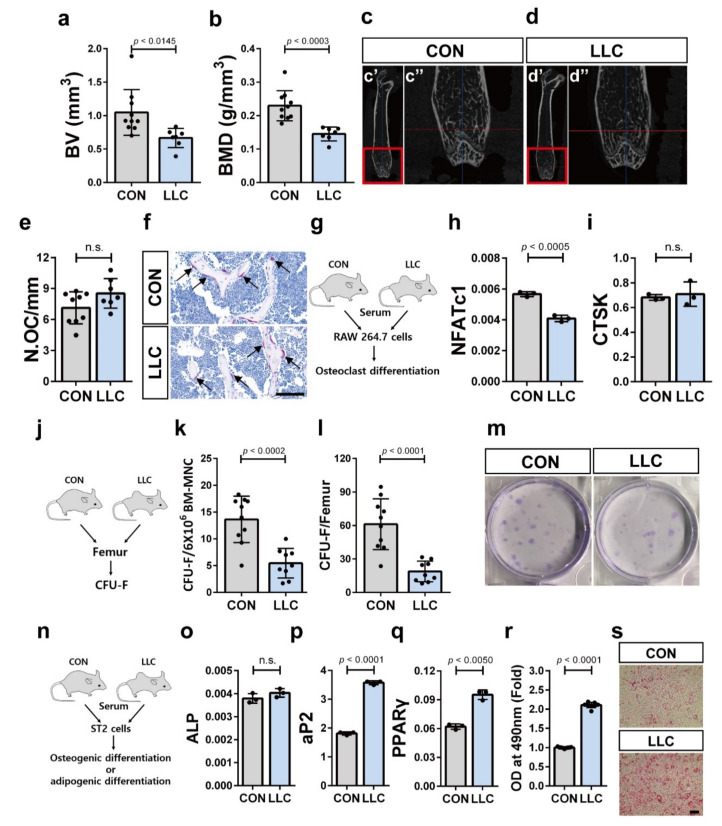
Bone loss in LLC cancer cachectic animal is associated with depletion and impaired differentiation balance of the bone marrow MSCs. (**a**,**b**) Trabecular bone volume (BV) and bone mineral density (BMD) were measured using femurs of LLC-bearing mice at 25 days postinoculation of LLC cells (LLC; *n* = 7 mice) or the control mice at 25 days post-sham-operation (CON; *n* = 10 mice). (**c**,**d**) Representative femoral microcomputed tomography (μCT) images from LLC-bearing mice (LLC) and the control (CON). The red boxed regions in c’ and d’ are magnified in c’’ and d’’. (**e**) Histological analysis of tartrate-resistant acid phosphatase (TRAP) stained sections obtained from femurs of LLC-bearing mice at 25 days postinoculation of LLC cells (LLC; *n* = 7 mice) or the control mice at 25 days post-sham-operation (CON; *n* = 9 mice). N.OC/mm, the number of osteoclasts/mm of bone surface. (**f**) Representative of TRAP stained section of the femurs. Arrow indicates TRAP-positive cells on trabecular bone (Scale bar, 100 μm). (**g**) Schematic representation of osteoclast differentiation assay of RAW 264.7 cells using differentiation induction media supplemented with serum isolated from LLC-bearing mice at 25 days postinoculation of LLC cells (LLC) or the control mice at 25 days post-sham-operation (CON). The expression levels of NFATc1 mRNA (**h**) and CTSK mRNA (**i**) were measured after osteoclast differentiation of RAW 264.7 cells. (**j**) Schematic representation of colony-forming unit-fibroblast (CFU-F) assay using MSCs isolated from femurs of LLC-bearing mice at 25 days postinoculation of LLC cells (LLC) or the control mice at 25 days post-sham-operation (CON). Frequency of CFU-F (**k**) or the number of CFU-F per femur (**l**) were analyzed in LLC cancer cachectic mice (*n* = 9 mice) or the control mice (*n* = 10 mice). (**m**) Representative images of CFU-F. (**n**) Schematic representation of osteogenic or adipogenic differentiation assay of ST2 cells using differentiation inducing media supplemented with serum isolated from LLC-bearing mice at 25 days postinoculation of LLC cells (LLC) or the control mice at 25 days post-sham-operation (CON). (**o**) The expression level of ALP mRNA was measured after the osteogenic differentiation of ST2 cells. The expression level of aP2 mRNA (**p**) and PPARγ mRNA (**q**) were measured after the adipogenic differentiation of ST2 cells. (**r**) The adipogenic differentiation of ST2 cells was also confirmed with Oil Red O staining. (**s**) Representative images of Oil Red O staining (Scale bar, 100 μm). Data are presented as the mean  ±  SD (*p* values were obtained by *t*-tests. n.s.; not significant).

It has been suggested that colon cancer cachexia is associated with bone loss [44]. Bone homeostasis is regulated by the integrated functional interaction between osteoclasts derived from HSCs and osteoblasts derived from MSCs inside of bone marrow [45,46,47]. LLC-bearing mice showing cancer cachexia phenotype also showed reduced trabecular bone volume and bone mineral density compared to the control mice (Figure 3a–d). We further examined whether cancer cachexia enhanced the differentiation of myeloid cells into osteoclasts since increased myeloid cell population along with bone loss phenotype was observed in LLC-bearing mice. Osteoclasts in LLC-bearing mice were identified by tartrate resistant acid phosphatase (TRAP) staining. TRAP staining of femur sections revealed that the number of osteoclasts on the bone surface did not significantly differ between the LLC-bearing mice and control mice (Figure 3e,f). As cancer cachexia is considered a systemic disease [48], the blood of LLC-bearing mice that contains factors derived both from distant tumor cells and normal cells in response to the tumor may systemically affect other tissues. We used RAW 264.7 cells to investigate this possibility. RAW 264.7 cells are monocyte/macrophage-like cells originating from BALB/c mice and have been used to study osteoclast differentiation. We treated RAW 264.7 cells under osteoclast differentiation condition with serum collected from LLC-bearing mice and tumor-free control mice. (Figure 3g) [49]. We then determined the expression level of nuclear factor-activated T cells c1 (NFATc1), a master regulator of osteoclast differentiation [50], and cathepsin K (CTSK), a phenotypic marker of osteoclast [51]. Incubation with serum from LLC-bearing mice caused slight downregulation of NFATc1 expression after induction of osteoclast differentiation compared to that when incubated with serum from control mice (Figure 3h). However, the expression level of CTSK after induction of osteoclast differentiation did not differ between cells incubated with serum from LLC-bearing mice and control mice (Figure 3i). These results suggest that bone loss associated with cancer cachexia is not due to the increased osteoclast differentiation. Then, we assessed whether the decline in the number of MSCs or the impaired differentiation of MSCs into osteoblasts contribute to the bone loss associated with cancer cachexia. To this end, we performed CFU-fibroblast (CFU-F) assays using BM-MNCs isolated from LLC-bearing mice on day 25 postinoculation of the LLC or control mice (Figure 3j). The frequency of CFU-F per 6 × 10^6^ BM-MNCs or femur of the LLC-bearing mice was lower than that of the control mice (Figure 3k–m). These results suggest that cancer cachexia decreased the population of MSCs inside of bone marrow as well as the stem cell characteristics (stemness) of the bone marrow MSCs. We also investigated whether cancer cachexia affects the differentiation of the bone marrow MSCs into osteoblasts. Serum from the LLC-bearing mice or tumor-free control mice was added to mouse bone marrow-derived MSCs-like ST2 cells under osteoblast differentiation conditions [52] (Figure 3n) and the expression of alkaline phosphatase (ALP), a marker of osteogenic differentiation [53], was observed. There was no difference in the expression level of ALP between ST2 cells treated with serum from cancer cachectic mice or control mice (Figure 3o). This suggests that cancer cachexia did not impair bone marrow MSCs differentiation into osteoblasts. Importantly, serum from the LLC-bearing mice enhanced the adipogenic differentiation of ST2 cells. ST2 cells treated with serum from cancer cachectic mice showed increased expression of the aP2 and peroxisome-proliferator-activated receptor γ (PPARγ), adipocyte differentiation markers [54] under adipogenic differentiation conditions [55], compared to ST2 cells treated with serum from control mice (Figure 3p,q). Oil red O staining also confirmed that the adipogenic differentiation of the ST2 cells increased in response to serum from cancer cachectic mice compared control mice (Figure 3r,s). Therefore, serum components from LLC-bearing mice were sufficient to enhance the adipogenic differentiation of the bone marrow MSCs. Overall, these results suggest that the bone loss in LLC-bearing cancer cachectic mice is associated with the decrease in bone marrow MSCs population as well as the impaired balance between the osteogenic and adipogenic differentiation of the bone marrow MSCs.

### 2.4. Cancer Cachexia Increases Adipogenic Differentiation of the Bone Marrow MSCs via Activation of JAK/STAT as Well as Glucocorticoid Signaling and Decreases Bone Marrow MSCs in a Glucocorticoid-Dependent Manner

**Figure 4:**
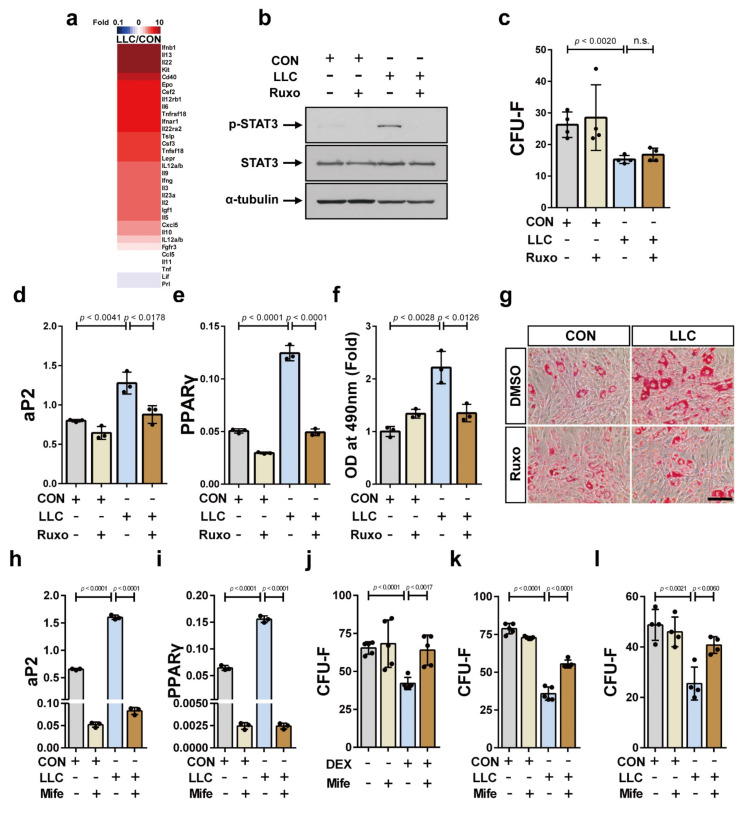
The activation of JAK/STAT and glucocorticoid signaling are associated with the depletion and the unbalanced differentiation capacity of bone marrow MSCs in LLC-cancer cachexia, respectively. (**a**) Protein content in the serum of LLC-bearing mice at 25 days postinoculation of LLC cells (LLC) and the control mice at 25 days post-sham-operation (CON) was determined using protein antibody arrays. Proteins related to JAK/STAT signaling activity were analyzed. Thirty-one proteins in the serum of LLC-bearing mice that showed a difference of at least 1.3 times compared to the value in the control (*p* value < 0.05) were presented. (**b**) Western blot analysis was performed using ST2 cells that had been treated with serum derived from LLC-bearing (LLC) or the control mice (CON) for 24 h after pretreatment with ruxolitinib (+ Ruxo) or solvent (- Ruxo) for 30 min. Uncropped Western blot images are available in Figure S2. (**c**) CFU-F assay was performed using ST2 cells that had been treated with serum derived from LLC-bearing mice (LLC) or the control mice (CON) for three days in addition of ruxolitinib (+ Ruxo) or solvent (- Ruxo). The expression levels of aP2 mRNA (**d**) and PPARγ mRNA (**e**) were analyzed after the adipogenic differentiation of ST2 cells using differentiation inducing media supplemented with serum derived from LLC-bearing mice (LLC) or the control mice (CON) in addition of ruxolitinib (+ Ruxo) or solvent (- Ruxo). (**f**,**g**) The adipogenic differentiation of ST2 cells was also confirmed with Oil Red O staining (Scale bar, 100 μm). The expression levels of aP2 mRNA (**h**) and PPARγ mRNA (**i**) were analyzed after the adipogenic differentiation of ST2 cells using differentiation inducing media supplemented with serum derived from LLC-bearing mice (LLC) or the control mice (CON) treated with mifepristone (+ Mife) or solvent (- Mife). (**j**) CFU-F assay was performed using ST2 cells that had been treated with dexamethasone (DEX, 1 μM) for 3 days with the addition of mifepristone (+ Mife) or solvent (- Mife). CFU-F assay was performed using ST2 cells (**k**) or human bone marrow MSCs (**l**) that had been treated with the culture media supplemented with serum derived from LLC-bearing mice (LLC) or the control mice (CON) for three days with the addition of mifepristone (+ Mife) or solvent (- Mife). Data are presented as the mean  ±  SD (*p* values were obtained by *t*-tests. n.s.; not significant).

We investigated the underlying molecular and cellular mechanisms by which the distant tumor cells affect the bone marrow-resident MSCs. We performed protein antibody array using the serum from LLC-bearing and control mice to identify factors that leads to the decrease in MSCs population in the bone marrow and the upregulation of their adipogenic differentiation. IL-6 is the major driver of cancer cachexia and activates the JAK/STAT signaling that is associated with muscle wasting in cancer cachexia [56]. Notably, factors including IL-6, IL-22, IFN-β, and granulocyte-macrophage colony-stimulating factor (GM-CSF), which activate the Janus kinase (JAK)/signal transducer and activator of transcription (STAT) pathway (Gene Ontology: 1904892) [57] were upregulated in the serum of the LLC-bearing mice compared to that in serum of the tumor-free control mice (Figure 4a and Figure S1). To confirm this finding, we determined STAT3 phosphorylation in serum-treated ST2 cells. STAT3 phosphorylation in response to the serum from the LLC-bearing mice was higher compared to that in response to control mice serum (Figure 4b). The increase in STAT3 phosphorylation was inhibited upon treatment with ruxolitinib, a potent and selective inhibitor of JAK1/2 with modest selectivity to JAK3 [58] (Figure 4b). The CFU-F frequency of the ST2 cells treated with the serum from the LLC-bearing mice was lower compared to that of the cells treated with serum from the control mice, likely because the MSCs derived bone marrow of the LLC-bearing mice showed lower CFU-F frequency than the MSCs derived from the control mice; however, the impaired CFU-F frequency was not rescued by ruxolitinib treatment (Figure 4c). Then, we examined the effect of ruxolitinib on the increased adipogenic differentiation of the ST2 cells treated with the serum from the LLC-bearing cancer cachectic mice. Pretreatment with ruxolitinib significantly inhibited the increase in aP2 and PPARγ expression in the cancer cathetic mice serum-treated ST2 cells under adipogenic differentiation conditions (Figure 4d,e). Oil red O staining confirmed that ruxolitinib decreased the adipogenic differentiation potential of these ST2 cells (Figure 4f,g). These results suggest that the CFU-F potential of the bone marrow MSCs in LLC-bearing mice is independent of JAK/STAT activation; in contrast, JAK/STAT activation in bone marrow MSCs directly regulates their adipogenic differentiation in response to cancer cachexia.

Next, we investigated the mechanism underlying the decrease in the CFU-F potential of the bone marrow MSCs in LLC-bearing cancer cachectic mice. The serum from LLC-bearing mice contains factors derived from both tumor cells and host cells in response to the tumor cell-derived factors. As IL-6 induces glucocorticoid secretion [2,13] and muscle wasting in cancer cachexia animal model reportedly depends on intact glucocorticoid signaling in skeletal muscle [15], we studied the role of glucocorticoid on bone marrow MSCs differentiation. Importantly, it has been demonstrated that glucocorticoid decreased MSCs pool in the femur of patients with osteonecrosis [33]. We examined if glucocorticoid signaling affects the differentiation capacity and/or CFU-F potential of the bone marrow MSCs in LLC-bearing cancer cachectic mice. Pretreatment with mifepristone, a glucocorticoid receptor antagonist [59], significantly inhibited the increase in the expression levels of aP2 and PPARγ in ST2 cells treated with the serum derived from LLC-bearing cancer cachectic mice under the adipogenic differentiation condition (Figure 4h,i). Dexamethasone, a potent synthetic glucocorticoid and one of the most effective anti-inflammatory drugs [30,31], decreased CFU-F frequency of ST2 cells, while mifepristone abrogated the effects of dexamethasone on CFU-F of ST2 cells (Figure 4j). Most importantly, the decrease in the colony forming capacity of the serum-treated ST2 cells was significantly rescued with mifepristone (Figure 4k). Similarly, the colony forming capacity of human bone marrow MSCs decreased in response to serum from LLC-bearing mice, and the decrease was rescued by mifepristone (Figure 4l). Overall, these results suggest that glucocorticoid systemically decreases CFU-F of the bone marrow MSCs and contributes to the skewing of the bone marrow MSCs differentiation toward adipogenic fate in LLC cancer cachexia.

## 3. Discussion

Cancer cachexia is considered a systemic inflammatory disease. Many organs, including the liver, brain, muscle, and fat tissue communicate with each other and influence the systemic inflammatory environment of cancer cachexia. Both tumor cells and activated immune cells secrete soluble factors, including proinflammatory cytokines, and synergistically work to form the systemic inflammation environment of cancer cachexia. The differentiation of the bone marrow HSCs into various immune cells is essential for systemic immune response and is tightly regulated by their microenvironment where various types of cells, including MSCs, reside. The bone marrow MSCs that can be differentiated into osteoblasts and adipocytes control the differentiation and maintenance of HSCs via various mechanisms. Therefore, the bone marrow HSCs may be affected by cancer cachexia intrinsically as well as extrinsically via a change in their niche components, namely the bone marrow MSCs. The mechanisms as well as the significance of skeletal muscle wasting and fat loss in cancer cachexia have been identified. However, whether bone metastasis-free tumor growth is associated with bone loss in cancer cachexia remains unclear [60]. Importantly, muscle loss is associated with bone dysfunction, which reduces mobility and quality of life [48,60] and this association was also found in cancer patients [61]. Therefore, it is important to determine whether tumor growth is associated with the occurrence of bone loss in cancer cachexia and identify mechanisms underlying bone loss. Bone loss was observed in animal models of colon cancer cachexia [44]. Here, we demonstrated that bone loss occurs in LLC-bearing mice, an animal model of lung cancer cachexia as well as muscle and fat loss. More importantly, we demonstrated that the differentiation capacity of the bone marrow MSCs is impaired via intrinsic activation of JAK/STAT in cancer cachexia. We further found that glucocorticoid signaling mediates the decrease in bone marrow MSCs population associated with bone loss in the cancer cachexia model.

The increase in myeloid lineage cells in the peripheral blood of cancer cachectic mice was accompanied by the reprogramming of hematopoiesis in the bone marrow. The number of the BM-MNCs increased before muscle and fat loss. The number of the hematopoietic progenitors also increased in the bone marrow of the cancer cachectic mice compared to the control mice, suggesting that the reprogramming of the hematopoiesis in the bone marrow may occur at the onset of cancer cachexia and results in systemic expansion of circulating myeloid lineage cells, neutrophils. Hematopoiesis reprogramming in the bone marrow of the cancer cachectic animal model may occur in two different ways—HSCs-intrinsically or HSCs-extrinsically via its microenvironment, HSC niche. The serum of the cancer cachectic animal model activated JAK/STAT signaling in bone marrow MSCs, one of the most important cellular microenvironmental factors for the bone marrow HSCs. Interestingly, the bone marrow MSCs were differentiated into adipocytes rather than osteoblasts under cancer cachexia environment and the activation of JAK/STAT contributed to the skewing of the bone marrow MSCs toward adipogenic fate. Glucocorticoid signaling also induced the skewing of the bone marrow MSCs differentiation. The bone marrow adipocyte is identified as a negative regulator of normal hematopoiesis [62,63]. An increase in its population inside the bone marrow is associated with certain clinical conditions, including osteoporosis, aging, and obesity, as well as clinical treatments, such as radiation, chemotherapy, and glucocorticoid treatment, all of which accompany myeloid skewing [62,63,64]. Therefore, the impaired differentiation balance of the bone marrow MSCs may cause hematopoiesis remodeling at the onset of cancer cachexia following an increase in the bone marrow adipocytes. In addition, we observed that JAK/STAT signaling was activated in hematopoietic precursor cell-7 (HPC-7), a murine bone marrow hematopoietic precursor cell line, in response to serum isolated from our cancer cachectic animal model (unpublished). Therefore, further experiments are required to verify whether JAK/STAT is intrinsically activated in bone marrow HSCs in response to cancer cachexia environment and thereby contributes to the hematopoiesis remodeling in the bone marrow. Although fat loss is one of the typical phenotypes in cancer cachexia, the differentiation of the bone marrow MSCs was interestingly skewed toward adipogenic fate in the present study. Further investigation is needed to determine if the bone marrow has more adipocytes in the cancer cachexia environment compared to that in the normal/control environment, and also elucidate the role of the bone marrow adipocytes in the cancer cachexia of the bone marrow.

The results of the present study demonstrated that bone loss is associated with lung cancer cachexia in the animal model. Furthermore, our findings suggest that bone loss is associated with the decrease as well as impaired differentiation balance of bone marrow MSCs. Further, JAK/STAT activation did not affect the colony-forming capacity of the bone marrow MSCs; however, it skewed their differentiation toward adipogenic fate. Interestingly, glucocorticoids, which are elevated in serum of cancer patients, including those suffering from cancer cachexia, reduced the ability of the bone marrow MSCs to form colonies. Thus, these results suggest that glucocorticoid signaling decreases the population of MSCs in the bone marrow and subsequently may induce the bone loss that reduces physical strength and quality of life in cancer cachexia patients. Currently, megestrol acetate, a synthetic progestin that can bind to the glucocorticoid receptor and induce glucocorticoid-like effects [65], is approved for appetite stimulation in cancer cachexia. However, the mechanisms by which megestrol acetate improves appetite are not known. The present results suggest that megestrol acetate treatment may exhibit potential side effects, bone loss and bone marrow MSCs depletion, in addition to other potential side effects, such as edema, which is in accordance with a previous report [66]. Despite LLC xenograft being a commonly used mouse model of cancer cachexia, it has its drawbacks. For example, it may not specifically mimic cancer cachexia and may lead to mixed outcomes of cachexia-free tumor and cancer cachexia. Further experiments would be required to verify that the current findings are specific to cancer cachexia.

## 4. Materials and Methods

### 4.1. Cell Culture

LLC1 (LLC) cell line was obtained from the American Type Culture Collection (ATCC) (Manassas, VA, USA) and maintained in Dulbecco’s modified Eagle’s medium/high glucose (DMEM/high; GE Healthcare Life Sciences, Logan, UT, USA) supplemented with 10% heat-inactivated fetal bovine serum (FBS; Invitrogen, Carlsbad, CA, USA) and 1% penicillin/streptomycin (P/S; Invitrogen). Murine bone marrow MSCs-like ST2 cell line was obtained from Riken cell bank (Tsukuba, Japan). ST2 cells were cultured with RPMI 1640 (Invitrogen) supplemented with 10% FBS and 1% P/S. ST2 cells between passage 5 to 7 were used for all experiments. Human bone marrow MSCs were obtained from LONZA (Basel, Switzerland) and cultured in Mesenchymal Stem Cell Growth Media (LONZA). Human bone marrow MSCs in passage 5 were used for all experiments. RAW 264.7 cells were obtained from the Korean Cell Line Bank (Korea, Seoul, South Korea) and maintained in DMEM/high supplemented with 10% FBS and 1% P/S.

### 4.2. Experimental Model of Cancer Cachexia

All animal experiments were approved by the animal experiment ethics committee of Kyung Hee Hospital Medical Center (KHMC-IACUC-2017-029) and performed in accordance with the Institutional Animal Care and Use Committee (IACUC) guidelines. Seven-week-old-male C57BL/6J mice (DBL, Seoul, South Korea) were used in all animal experiments. Mice were anesthetized by intraperitoneal injection of xylazine (16 mg/kg) and ketamine (200 mg/kg) mixture (1:1). Following this, LLC cells in passage 5 (5 × 10^6^ cells in 150 µL phosphate buffered saline, PBS) were injected subcutaneously into the right flank. Control mice were injected with PBS (sham operation). Body weight was measured every five days. Tumor was carefully resected and weighed every five days to calculate tumor-free body weight as the body weight minus the tumor weight. After 0, 5, 15, 20, or 25 days, tumor, tibia muscles (gastrocnemius, tibialis anterior, extensor digitorum longus, and soleus muscle), inguinal adipose tissue, and the spleen were also resected and weighed. On day 25 postinoculation of LLC cells, blood samples were collected in ethylene diaminetetraacetic acid (EDTA)-containing microtubes (BD, NJ, USA) to analyze the complete blood counts using ADVIA^®^ 2120 Hematology System (SIEMENS Healthineers, Erlangen, Germany).

### 4.3. Enzyme-Linked Immunosorbent Assay (ELISA)

IL-6 level in LLC-bearing mice serum was analyzed using ELISA antibody kit (R&D system Inc., Minneapolis, MN, USA) according to manufacturer’s instructions. Blood samples were collected from LLC-bearing and control mice on 15, 20, and 25 days postinoculation of LLC cells and post-sham operation, respectively, and centrifuged to obtain serum.

### 4.4. Protein Antibody Array

Serum samples from control and LLC-bearing mice were analyzed using RayBio biotin label-based mouse antibody array (L-308; RayBiotech, Norcross, GA, USA) according to manufacturer’s instructions. Blood was collected from the LLC-bearing and control mice on day 25 postinoculation of LLC cells and sham operation, respectively, and processed to obtain serum that was stored at −80 °C until further use. Serum samples of nine experimental mice with cancer cachexia and 10 control mice were combined to generate pooled serum sample sets. For each array, protein intensity values were background subtracted and scaled using the internal control. Fold change in serum values from LLC-bearing mice versus those from control mice was calculated and the resulting values were presented in a heat map.

### 4.5. Serum Preparation for In Vitro Experiments

Serum samples from LLC-bearing mice (*n* = 15 mice) on day 25 postinoculation of LLC cells and tumor-free control mice (*n* = 15 mice) on day 25 were pooled to create two sets. These pooled serum sets were aliquoted and stored at −80 °C until further use. These sera were added to the culture media, used for in vitro experiments. The media supplemented with serum was filtered through a 0.2-micrometer filter (CORNING, Corning, NY, USA) before use.

### 4.6. Flow Cytometry

BM-MNCs were obtained by flushing the femurs with 1 mL PBS containing 1% FBS and 0.02% sodium azide using a 23-gauge needle (BD Biosciences, San Jose, CA, USA). The cells were filtered through a 40-micrometer filter (BD Biosciences), washed with PBS supplemented with 1% FBS and 0.02% sodium azide, and diluted to a density of 1 × 10^7^ cells/mL. Monoclonal antibodies against c-Kit (Biolegend, San Diego, CA, USA) and Sca1 (Biolegend) and a cocktail of biotin-labeled monoclonal antibodies against CD3, B220, Ter119, Gr1, and CD19 (STEMCELL Technologies, Vancouver, BC, Canada) were used for flow cytometry analysis. Flow cytometry was performed using BD FACSCalibur (BD Biosciences).

### 4.7. CFU Assay

Flushed BM-MNCs were plated in duplicate in 35-mm dishes (1.5 × 10^4^ cells/dish) using methylcellulose-based media (M3434, STEMCELL Technologies) according to the manufacturer’s instructions. After incubation for 10 days, all colonies were counted using an Eclipse TS100 light microscope (Nikon Instruments Inc., Melville, NY, USA). CFU frequency and CFU yield were expressed as the number of colonies per 1.5 × 10^4^ cells and the number of colonies divided by the number of BM-MNCs isolated from each femur, respectively.

### 4.8. Ex Vivo Microcomputed Tomography (μ-CT) Imaging

Femurs resected from control mice and LLC-bearing mice on day 25 postinoculation of LLC cells and sham operation, respectively, were fixed with 4% paraformaldehyde (PFA; 3M Science, Saint Paul, MN, USA). The entire length of the femurs were scanned using a μ-CT scanner (SkyScan 1173; Bruker, MA, USA) under the following conditions: 90 kV, 88 μA, 1.0 mm Al filter, 500 ms exposure, and 11.01 μm image pixel size. After three-dimensional reconstruction of bone images, trabecular bone volume and bone mineral density were analyzed in a 2-mm region of secondary spongiosa beginning at 0.5 mm below the growth plate.

### 4.9. Tartrate-Resistant Acid Phosphatase (TRAP) Staining

Femurs resected from control mice and LLC-bearing mice on day 25 postinoculation of LLC cells and sham operation, respectively, were fixed with 4% PFA (3M Science) for 7 days. Then, the femurs were decalcified in 14% EDTA (pH 7.0) and embedded in paraffin. Then, 4.0-micrometer-thick sections were prepared using Leica RM2255 microtome (Leica Biosystems, Wetzlar, Germany) and used for TRAP staining (GENOSS, Suwon, Korea) and hematoxylin counterstaining (GENOSS, Suwon, Korea). The stained sections were observed and images were captured using Pannoramic 250 digital slide scanner (3D Histech, Hungary). TRAP-positive multinucleated osteoclasts in the metaphyseal area, located 0.5 mm proximal to the growth plate and 0.5 mm away from the intracortical surface, were counted and the number of TRAP-positive multinucleated osteoclasts normalized to the bone perimeter were analyzed for each sample. The bone perimeter was measured using ImageJ software v 1.53e (NIH, Bethesda, MD, USA).

### 4.10. CFU-F Assay

BM-MNCs flushed from control and LLC-bearing mice on day 25 postinoculation of LLC cells and sham operation, respectively, were seeded in 6-well-plates at a density of 6 × 10^6^ cells/well using Minimum Essential Medium Eagle-Alpha Modification (α-MEM, Sigma, St. Louis, MO, USA) supplemented with 20% FBS, 1% P/S, 1% L-glutamine (Invitrogen), and 5% pyruvate (Invitrogen). After incubation for 14 days, the plates were washed with PBS three times and fixed with methanol for 10 min. Colonies were stained with 0.5% crystal violet solution (Sigma) for 2 h and counted using SZ2-ST stereomicroscope (Olympus corporation, Tokyo, Japan).

ST2 cells were seeded in normal culture media and cultured overnight. Then, the media were replaced with RPMI 1640 supplemented with 10% (*v/v*) serum isolated from LLC-bearing or control mice. After incubation for three days, ST2 cells were inoculated in 60-mm culture dish at a density of 1.3 × 10^3^ or 2.7 × 10^3^ cells/dish in normal culture media. Colony formation was evaluated after seven days. If necessary, ST2 cells were pretreated with 100 nM ruxolitinib (Caymanchemical, Ann Arbor, MI, USA) or 10 µM mifepristone (Sigma) for 30 min before culturing with mice serum containing media; ruxolitinib or mifepristone were added daily to the culture media during experimental period (3 days). To observe effects of dexamethasone on CFU-F of ST2 cells, ST2 cells were treated with 1 μM dexamethasone (Sigma) with or without mifepristone for 3 days. Then, the ST2 cells were seeded for CFU-F assay.

Human bone marrow MSCs were seeded in normal culture media and cultured overnight. Then, the media were replaced with DMEM/low glucose supplemented with 10% serum isolated from control or LLC-bearing mice. If required, human bone marrow MSCs were pretreated with 10 µM mifepristone for 30 min. Then, human bone marrow MSCs were seeded in 100-mm culture dishes at a density of 2 × 10^3^ cells/dish in DMEM/low glucose supplemented with 10% FBS, 1% L-glutamine, and 1% P/S. After incubation for 14 days, the colonies were counted.

### 4.11. Osteoclast Differentiation

RAW264.7 cells were plated in 24-well plates at a density of 1.6 × 10^3^ cells/cm^2^ in DMEM supplemented with 100 ng/mL RANKL (R&D system), 7.5% FBS, and 2.5% (*v/v*) serum from control or LLC-bearing mice. The cells were incubated for six days and the media were replaced with fresh media every three days.

### 4.12. Osteoblast Differentiation

ST2 cells were plated in 24-well plates at a density of 1 × 10^4^ cells/cm^2^ in DMEM supplemented with 10 mM β-glycerophosphate (Sigma), 50 µg/mL ascorbic acid (Sigma), 10% FBS, 1% P/S, and 10% (*v*/*v*) serum from control or LLC-bearing mice. The cells were incubated for 10 days and the media were replaced with fresh media every three days.

### 4.13. Adipocyte Differentiation and Oil Red O Staining

ST2 cells were plated in 6-well plates at a density of 1 × 10^4^ cells/cm^2^ in normal culture media. After 24 h, the media were replaced with media supplemented with 0.5 mM 3-isobutyl-1-methylxanthine (IBMX, Sigma), 1 nM dexamethasone (Sigma), 10 µg/mL insulin (Sigma), and 10% (*v*/*v*) serum from control or LLC-bearing mice. After incubation for three days, the cells were washed with PBS twice and fixed with 4% PFA in PBS for 1 h. The fixed cells were washed with water twice and incubated with 60% isopropanol (Sigma) for 5 min. Then, the cells were stained with 0.3% Oil Red O (Sigma) for 30 min. After washing the plate twice with water, images of the stained cells were captured using an Eclipse TS100 light microscope (Nikon). To quantify the Oil Red O stained lipid drops, the stained cells were rinsed with 60% isopropanol and subsequently dried. The stains were eluted with 1 mL of 100% isopropanol for 10 min at 25 °C and the optical density was measured at 540 nm using an absorbance plate reader (Spectramax190; Molecular Devices; Thermo Fisher Scientific Inc., Waltham, MA, USA). If required, 100 nM ruxolitinib or 10 µM mifepristone was added daily for three days during which the ST2 cells underwent adipogenic differentiation on incubation with the mice serum.

### 4.14. Western Blot Analysis

ST2 cells were seeded in 12-well plates at a density of 1 × 10^4^ cells/cm^2^ in normal culture media. After 24 h, the cells were incubated with FBS-free DMEM/high supplemented with 1% P/S for 18 h. Then, the media were replaced with FBS-free DMEM/high supplemented with 1% P/S and 10% (*v*/*v*) serum from control or LLC-bearing mice and the cells were incubated for another 24 h. If required, the cells were pretreated with 100 nM ruxolitinib or DMSO for 30 min before adding the new media. In order to obtain total cell lysates, the cells were washed twice with ice cold PBS. Then, they were incubated with 120 µL 2× SDS buffer (100 mM Tris-HCl [pH 6.8], 2% [*w/v*] SDS, 0.01% bromophenol blue, 20% glycerol, and 10% β-mercaptoethanol) for 5 min at 25 °C. The cells were collected by scraping using cell scrapers, lysed, and proteins were denatured by incubating at 95 °C for 5 min. Western blot analysis was performed using primary antibodies against phospho-STAT3 (1:500; Cell Signaling Technology, Danvers, MA, USA), STAT3 (1:1000; Cell Signaling Technology), and α-tubulin (1:30,000, Sigma) according to standard protocols.

### 4.15. Quantitative Real-Time Polymerase Chain Reaction (qRT-PCR)

Total RNA was extracted from the cells using TRIzol reagent (Invitrogen) and cDNA, was synthesized using Superscript III first-strand synthesis kit (Invitrogen). qRT-PCR was performed using Power SYBR Green PCR Master Mix (Invitrogen). Mouse 36B4 *Rplp0* gene was used as an internal control. The following primers were used to detect the expression of the control and target genes: *Rplp0* (sense): 5′-GAA CATCTCCCCCTTCTCCTT-3′, *Rplp0* (antisense): 5′-GCAGGGCCTGCTCTGTGAT-3′; *Nfatc1* (sense): 5′- AAAGGAGAGGTCGGACTCGG-3′, *Nfatc1* (antisense): 5′- AACTGTAGTGTTCTT CCTCGG C-3′; *Ctsk* (sense): 5′- GCCACGCTTCCTATCCGAAA-3′, *Ctsk* (antisense): 5′- CGAGAGATTTCATCCACCTTGC-3′; *Fabp4* (sense): 5′- CATGGCCAAGCCCAACAT-3′, *Fabp4* (antisense): 5′- CGCCCAGTTTGAAGGAAATC-3′; *Pparg* (sense): 5′- CGCTGATGCACTGCCTATGA-3′, *Pparg* (antisense): 5′- AGAGGTCCACAGAGCTGATTCC-3′; *Alpl* (sense): 5′- CCAACTCTTTTGTGCCAGAGA-3′, and *Alpl* (antisense): 5′- GGCTACATTGGTGTTGAGCTTTT-3′.

### 4.16. Statistical Analysis

Data were expressed as mean or mean ± SD. Statistical significance was analyzed using two-way ANOVA and two-tailed Student’s t-tests. GraphPad version 6.07 (GraphPad Software Inc., San Diego, CA, USA) was used for statistical analysis. *p* values < 0.05 were considered significant.

## 5. Conclusions

The current study provides evidence that cancer cachexia environment decreases the population of bone marrow MSCs via glucocorticoid signaling and impairs the fine balance of osteogenic and adipogenic differentiation of the MSCs via the activation of JAK/STAT as well as glucocorticoid signaling. This alteration in MSCs of cancer cachexia bone marrow is associated with bone loss and myeloid skewing in the circulation. These results suggest that the impaired function of the bone marrow MSCs may cause bone loss and pathological differentiation of the bone marrow HSCs under cancer cachexia. A further understanding of the multifaceted mechanisms underlying the functional interaction between MSCs and HSCs in cancer cachexia bone marrow is vital for providing novel therapeutic strategies for cancer cachexia.

## Data Availability

The data presented in this study are available in the article and supplementary material.

## Supplementary Materials

The following are available online at https://www.mdpi.com/2072-6694/13/5/1059/s1, Figure S1: Results of protein antibody array:A list of proteins that regulate JAK/STAT signaling, Figure S2: The original blot images of Figure 4b.
