# Supplementary material for: Bone Marrow Homeostasis Is Impaired via JAK/STAT and Glucocorticoid Signaling in Cancer Cachexia Model
Source: Cancers (Basel). 2021 Mar 2;13(5):1059. doi: 10.3390/cancers13051059 (PMC7958949; doi:10.3390/cancers13051059)
Supplement: Supplementary file 1 [file cancers-13-01059-s001.pdf]

Bone Marrow Homeostasis is Impaired via JAK/STAT and Glucocorticoid Signaling in Cancer Cachexia Model

Jinyeong Yu [1,†], Sanghyuk Choi [1,†], Aran Park [1], Jungbeom Do [2], Donghyun Nam [2], Youngjae Kim [2], Jinok Noh [2], Kil Yeon Lee [3], Chi Hoon Maeng [4] and Ki-Sook Park [2,5,*]

[1] Graduate School of Biotechnology, Kyung Hee University, Yongin 17104, Korea
[2] Department of Biomedical Science and Technology, Graduate School, Kyung Hee University, Seoul 02447, Korea
[3] Department of Surgery, College of Medicine, Kyung Hee University, Seoul 02447, Korea
[4] Department of Internal Medicine, College of Medicine, Kyung Hee University, Seoul 02447, Korea
[5] East-West Medical Research Institute, Kyung Hee University, Seoul 02447, Korea

* Correspondence: Ki-Sook Park, PhD, kisookpark@khu.ac.kr, Tel.: +82-2-958-9368
[†] equally contributing authors

Gene symbol	Antibody Name	LLC 25d /CTL 25d
Ctf1	Cardiotrophin-1	NA
Ghr	Growth Hormone R	NA
Il4	IL-4	NA
Il6ra	IL-6 R	NA
Il10ra	IL-10 R alpha	NA
Il15	IL-15	NA
Il21	IL-21	NA
Il24	IL-24	NA
Il31ra	IL-31 RA	NA
Lep	LEPTIN(OB)	NA
Tnfrsf1a	TNF RI/TNFRSF1A	NA
Ifnb1	IFN-beta	48.133
Il13	IL-13	25.630
Il22	IL-22	19.904
Kit	SCF R/c-kit	19.543
Cd40	CD40	9.658
Epo	Erythropoietin (EPO)	7.400
Csf2	GM-CSF	6.851
Il6	IL-6	6.198
Tnfrsf18	GITR	5.911
Il22ra2	IL-22BP	5.157
Tslp	TSLP	4.681
Tnfsf18	GITR Ligand/TNFSF18	4.089
Ifng	IFN-g	2.739
Il3	IL-3	2.637
Il23a	IL-23	2.597
Il2	IL-2	2.467
Igf1	IGF-I	2.131
Il5	IL-5	2.042
Cxcl5	LIX	1.838
Il10	IL-10	1.811
Fgfr3	FGF R3	1.448
Ccl5	RANTES	1.199
Tnf	TNFalpha	0.853
Lif	LIF	0.798
Prl	Prolactin	0.739
Il11	IL-11	0.929
Csf3	G-CSF	4.614
Thpo	TPO	NA
Lepr	Leptin R	3.868
Il7	IL-7	NA
Il9	IL-9	2.876
Ifnar1	IFN-alpha/beta R1	5.418
Ifnar2	IFN-alpha/beta R2	NA
Il12rb1	IL-12 R beta 1	6.755
IL12a/b	IL-12p40/p70	2.971
IL12a/b	IL-12p70	1.528

Supplementary Figure. S1: Results of Protein antibody array: A list of proteins that regulate JAK/STAT signaling.

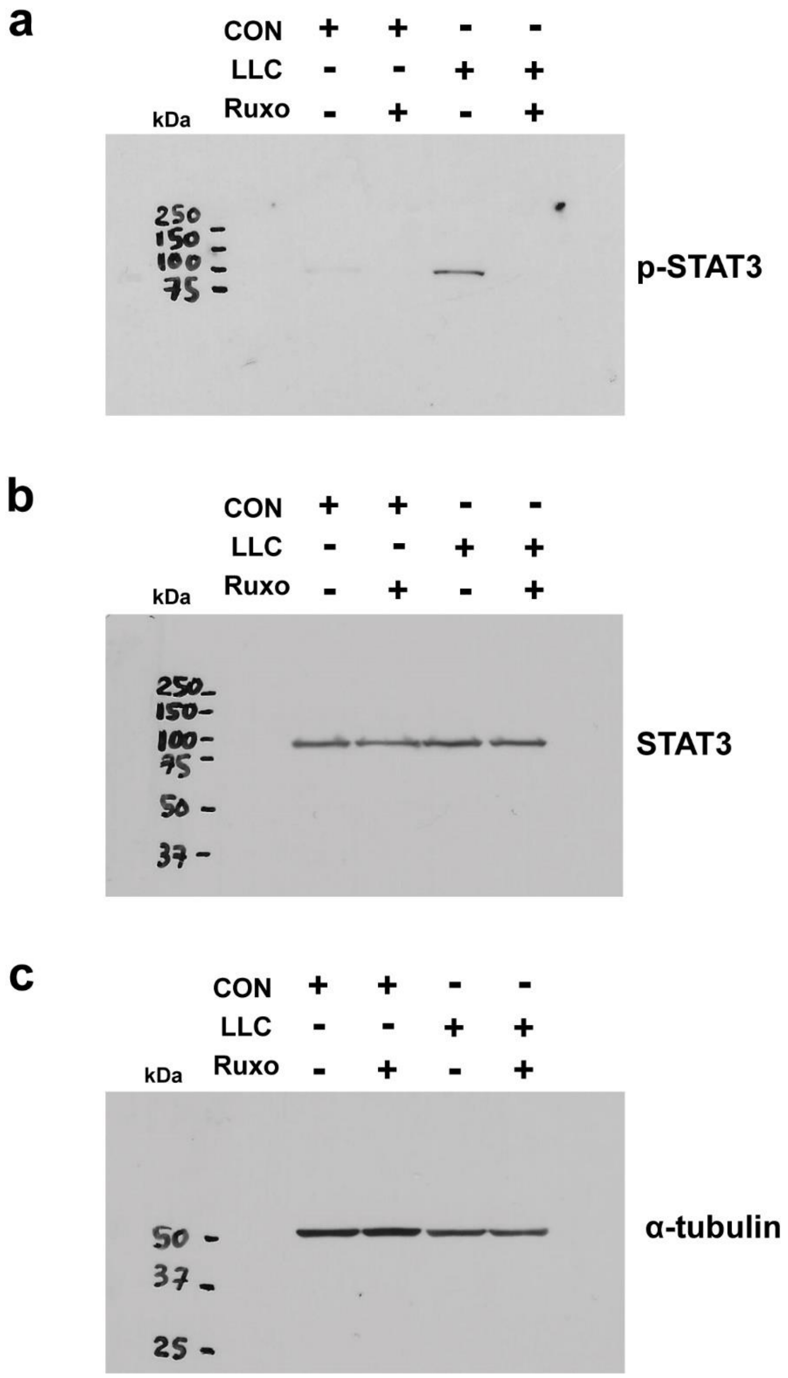

Supplementary Figure. S2: Full unedited blots of Figure 4b. (**a**) A full unedited blot for p-STAT3 (**b**) A full unedited blot for STAT3. (**c**) A full unedited blot for α-tubulin.
